# Requirement for Zebrafish Ataxin-7 in Differentiation of Photoreceptors and Cerebellar Neurons

**DOI:** 10.1371/journal.pone.0050705

**Published:** 2012-11-30

**Authors:** Constantin Yanicostas, Elisa Barbieri, Masahiko Hibi, Alexis Brice, Giovanni Stevanin, Nadia Soussi-Yanicostas

**Affiliations:** 1 INSERM, U676, Hôpital Robert Debré, Paris, France; 2 Université Paris Diderot, Sorbonne Paris Cité, Paris, France; 3 INSERM, U975, Paris, France; 4 Université Pierre et Marie Curie-Paris 6, Centre de Recherche de l'Institut du Cerveau et de la Moelle épinière, UMR_S975, GHU Pitié-Salpêtrière, Paris, France; 5 CNRS, UMR7225, Paris, France; 6 Laboratory for Vertebrate Axis Formation, RIKEN Center for Developmental Biology, Kobe, Hyogo, Japan; 7 Bioscience and Biotechnology Center, Nagoya University, Nagoya, Japan; 8 Ecole Pratique des Hautes Etudes, Paris, France; Institute of Genetics and Molecular and Cellular Biology, France

## Abstract

The expansion of a polyglutamine (polyQ) tract in the N-terminal region of ataxin-7 (atxn7) is the causative event in spinocerebellar ataxia type 7 (SCA7), an autosomal dominant neurodegenerative disorder mainly characterized by progressive, selective loss of rod-cone photoreceptors and cerebellar Purkinje and granule cells. The molecular and cellular processes underlying this restricted neuronal vulnerability, which contrasts with the broad expression pattern of atxn7, remains one of the most enigmatic features of SCA7, and more generally of all polyQ disorders. To gain insight into this specific neuronal vulnerability and achieve a better understanding of atxn7 function, we carried out a functional analysis of this protein in the teleost fish *Danio rerio*. We characterized the zebrafish *atxn7* gene and its transcription pattern, and by making use of morpholino-oligonucleotide-mediated gene inactivation, we analysed the phenotypes induced following mild or severe zebrafish atxn7 depletion. Severe or nearly complete zebrafish atxn7 loss-of-function markedly impaired embryonic development, leading to both early embryonic lethality and severely deformed embryos. More importantly, in relation to SCA7, moderate depletion of the protein specifically, albeit partially, prevented the differentiation of both retina photoreceptors and cerebellar Purkinje and granule cells. In addition, [1–232] human atxn7 fragment rescued these phenotypes showing strong function conservation of this protein through evolution. The specific requirement for zebrafish atxn7 in the proper differentiation of cerebellar neurons provides, to our knowledge, the first *in vivo* evidence of a direct functional relationship between atxn7 and the differentiation of Purkinje and granule cells, the most crucial neurons affected in SCA7 and most other polyQ-mediated SCAs. These findings further suggest that altered protein function may play a role in the pathophysiology of the disease, an important step toward the development of future therapeutic strategies.

## Introduction

SCA7 is an autosomal dominant neurodegenerative disorder caused by the expansion of a translated CAG repeat in the *SCA7/ataxin-7 *gene, leading to expansion of a polyQ tract located in the N-terminal region of the encoded protein, ataxin-7 (atxn7) [1]. SCA7 thus belongs to the family of polyQ expansion disorders, also named polyQ diseases, a group of neurodegenerative disorders comprising spinobulbar muscular atrophy (SBMA) [2], Huntington’s disease (HD) [3], dentatorubral-pallidoluysian atrophy (DRPLA) [4] and spinocerebellar ataxia (SCA) 1, 2, 3, 6, 7, and 17 [1], [5]–[13].

All polyQ diseases are characterized by progressive, selective degeneration of distinct, albeit disease-specific, neuronal populations. Vulnerable neurons in SCA7 include Purkinje cells, a neuronal population that is affected in most polyQ-mediated SCAs, excepted SCA3 [14], and several other neuronal populations such as cerebellar granule cells, neurons of inferior olive and cranial nerve nuclei, and also rod-cone photoreceptors, a cell population that is spared in other SCA types [15]–[17]. Beside this disease-specific neuronal vulnerability, all polyQ disorders share several common features: (*i*) progressive neuronal dysfunction and degeneration, (*ii*) expression of the disease phenotype when the size of the polyCAG/polyQ expansion reaches a precise threshold, which varies according to the gene, (*iii*) a strong negative correlation between age at onset and size of the polyQ tract, (*iv*) instability of the CAG repeat during transmission, with a strong tendency to expansion, resulting in an effect called anticipation (cf. [18]–[22]). Paradoxically, apart from their polyQ tract, the disease proteins display neither structural nor functional similarities.

Atxn7 is a subunit of a multiprotein complex, the Spt-Ada-Gcn5-acetyltransferase (SAGA) complex, which is involved in histone acetylation and transcription regulation [23]–[26]. A body of work on several mouse models has demonstrated that rod-cone photoreceptor degeneration in SCA7 is at least partially a consequence of interference of polyQ-expanded atxn7 with CRX, a homeodomain protein that plays a key role for proper transactivation of photoreceptor genes [27]–[30]. By contrast, the molecular and cellular bases of the selective vulnerability of other neuronal populations, such as cerebellar Purkinje cells, remain poorly understood. In mammals, challenging the specific neuronal loss, the *atxn7* gene is, like almost all the genes underlying polyQ disorders, expressed in numerous neuronal populations, including neurons, which are spared in SCA7, but also in a large set of non-neuronal tissues, [16], [31], [32].

To further address this issue, a better understanding of the normal function of atxn7 could provide important insights. However, though the group of Zoghbi generated an *atxn7* KO mice line [33], the phenotype of these mice has not yet been described. Here, we show that the *D. rerio atxn7* gene was broadly expressed throughout development from the one-cell stage onward, although in adults it was transcribed in several neuronal populations, including granule, but not Purkinje cells. Loss of function experiments demonstrated that severe depletion of zebrafish atxn7 impaired early development, leading to embryonic lethality combined with highly deformed embryos. Significantly, in relation to the disease, moderate depletion of the protein specifically compromised the differentiation of photoreceptors and cerebellar Purkinje and granule cells, the main crucial neuronal populations that are affected in SCA7. These findings lend new insight into the specific vulnerability of cerebellar neurons in SCA7 and also suggest that altered ataxin-7 function may play a role in the disease process.

## Results

### Characterization of the Zebrafish *atxn7* gene

**Figure 1:**
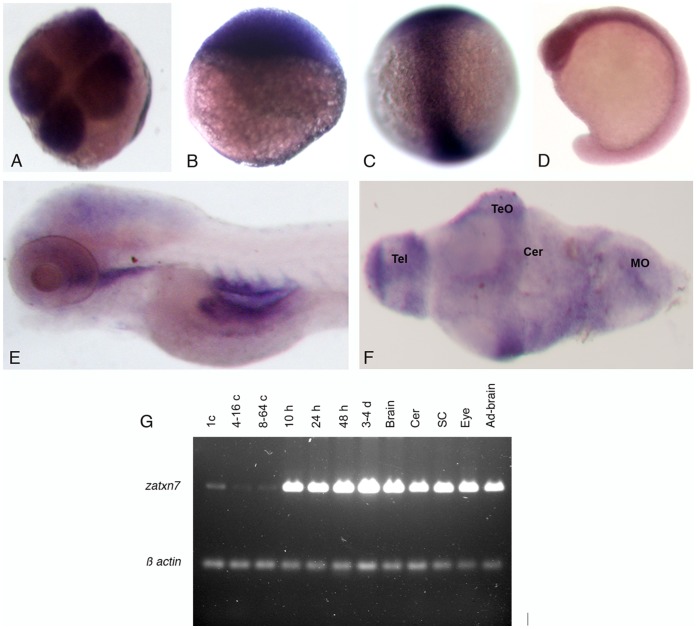
Transcription of the zebrafish *atxn7* gene during development. (A-F) *In situ* detection of zebrafish *atxn7* transcripts on either whole mount embryos at the four-cell stage (A), or 3 (B), 8 (C), 16 (D), and 48 hpf (E) or dissected brain of 120 hpf embryos (F). (I) RT-PCR analysis of zebrafish *atxn7* transcript accumulation in 1- (1 c), 4- to 16- (4–16 c), and 8- to 64-cell embryos (8–64 c), or 10 (10 h), 24 (24 h), 48 (48 h) and 72 to 96 hpf embryos (3–4 d) and dissected adult brain (Brain), cerebellum (Cer), spinal cord (SC), eye (Eye) and remaining tissues (Ad-brain). RT–PCR for *β-actin* is shown as a positive control. Abbreviations: Cer, cerebellum; MO, medulla oblongata; TeO, tectum opticum; Tel, telencephalon.

**Figure 2:**
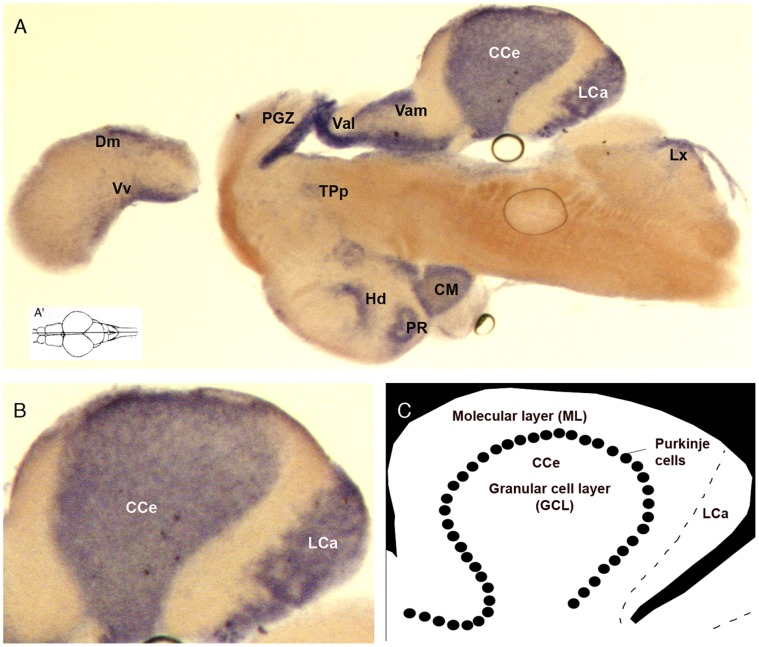
Transcription of the zebrafish *atxn7* gene in adult brain sections. *In situ* detection of zebrafish *atxn7* transcripts on midsagittal section of adult brain (A). Dorsal view of the adult zebrafish brain showing the position level of the parasagittal section shown in A (A’). A magnified view of the section showed in panel A (B). Schematic representation of zebrafish cerebellum (C). Anterior is to the left. Abbreviations: CC, crista cerebellaris; CCe, corpus cerebelli; CM, corpus mamillare; Dm, medial zone of dorsal telencephalic area; Hd, dorsal zone of the periventricular hypothalamus; LCa, lobus caudalis cerebelli; LX, lobus vagus; MON, medial octavolateralis nucleus; PGZ, periventricular grey zone; PR, posterior recess of diencephalic ventricle; TPp, periventricular nucleus of the posterior tuberculum; Val, lateral division of valvula cerebelli; Vam, medial division of valvula cerebellaris; Vv, ventral nucleus of the ventral telencephalon.

To identify the *Danio rerio atxn7* gene, we performed a blast analysis of the release Zv9 of the zebrafish genome sequence for genes showing sequence similarities with human *atxn7.* Our results identified 4 *atxn7* paralogs in zebrafish (Figure S1A), which are expressed in 24, 48 and 72 hours post-fecundation (hpf) embryos (Figure S1B and Figure 1G). However, molecular phylogeny deduced from ClustalW2 analysis showed that a single and *bona fide* ortholog of the human *atxn7* (*hatxn7*) gene is found in *D. rerio* (Figure S1A). This gene is referred to hereafter as zebrafish *atxn7* (*zatxn7*) (Ensembl ENSDARG00000074804). Sequencing of RT-PCR fragments encompassing the complete protein-coding region and part of the 5′ and 3′ UTRs showed that the zebrafish *atxn7* mRNA comprises 12 exons and encodes an 866 amino acid protein (Figure S1C) referred to hereafter as zebrafish atxn7. At the amino acid levels, the protein displayed 51.1 and 49.8% identities and 65.9 and 64.6% similarities compared with human and mouse atxn7, respectively. RT-PCR demonstrated that zebrafish *atxn7* transcripts were expressed at low levels in 1-, 4- to 8- and 16- to 64-cell embryos and at higher levels in embryos aged 10, 24, 48 and 72 hpf (Figure 1G). In dissected adult tissues, zebrafish *atxn7* RNAs were found in the brain, cerebellum, spinal cord, eye and non-neuronal tissues (Figure 1G). RNA *in situ* hybridization revealed a uniform accumulation of transcripts in 4-cell and 3, 8 and 16 hpf embryos (Figure 1A–1D). High levels of zebrafish *atxn7* transcription were detected in the brain of 24 hpf embryos (Figure 1E). In the dissected brain of 120 hpf embryos, zebrafish *atxn7* mRNAs were found in various regions, including the anterior region of the telencephalon, optic tectum and cerebellum (Figure 1F). On adult brain sections, zebrafish *atxn7* mRNAs accumulated in several neuronal populations (Figure 2A) including cerebellar granule cells, but not Purkinje cells (Figure 2B).

### Zebrafish atxn7 Plays an Essential Role for Embryo Development

**Figure 3:**
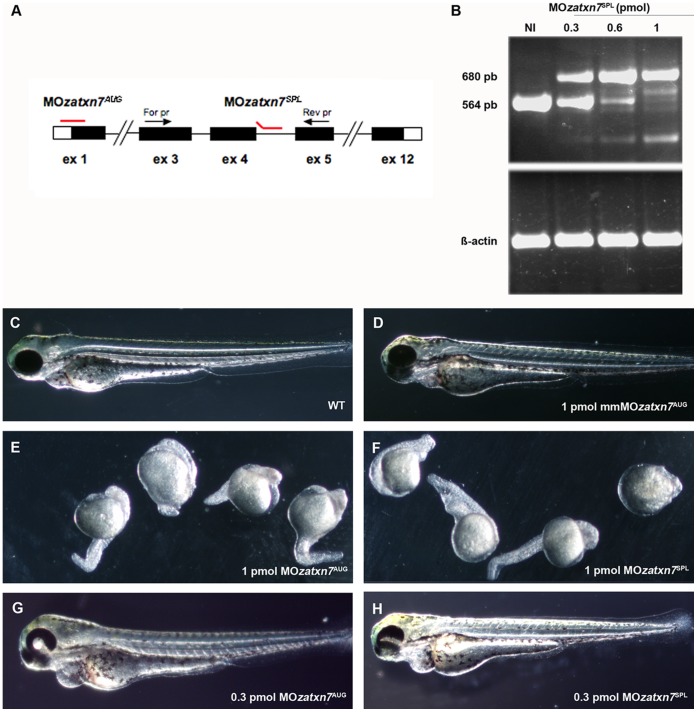
Morpholino-mediated inactivation of zebrafish *atxn7* impairs embryonic development. Schematic representation of the zebrafish *atxn7* gene showing exons 1 (ex 1), 3 to 5 (ex 3–5) and 12 (ex 12) (black boxes), location of MO*zatxn7^AUG^* and MO*zatxn7^SPL^* (red lines) and position of oligonucleotides (black arrows) used for RT-PCR analysis of MO*zatxn7^SPL^*-mediated inhibition of zebrafish *atxn7* intron 4 splicing (black arrows) (A). Untranslated exonic regions and intronic sequences are depicted as empty boxes and single lines, respectively. RT-PCR analysis of zebrafish *atxn7* intron 4 splicing in non-injected (NI) and morphant embryos that had received 0.3, 0.6 and 1 pmol MO*zatxn7^SPL^* (B). RT-PCR for *β-actin* is shown as a positive control. Phenotypes of 48 hpf wild-type embryo (C), and age-matched 1 pmol mmMO*zatxn7^AUG^* (D), 0.3 pmol MO*zatxn7^AUG^* (G) and 0.3 pmol MO*zatxn7^SPL^* morphants (H). Phenotypes of 24 hpf 1 pmol MO*zatxn7^AUG^* (E) and 1 pmol MO*zatxn7^SPL^* morphants (F).

To gain insight into zebrafish atxn7 function, we made use of morpholino-oligonucleotide (MO)-mediated gene knockdown to investigate the phenotypes caused by various levels of zebrafish atxn7 depletion in embryos. First, we microinjected wild-type zebrafish embryos of the AB strain (referred to below as morphants) with MO*zatxn7^AUG^*, a MO designed to inhibit translation of zebrafish *atxn7* mRNA (Figure 3A). Injection of 1 pmol MO*zatxn7^AUG^* induced high percentages of embryonic lethality, with 24.8% and 60% of morphants dying before 10 and 24 hpf, respectively (*n*  = 165) (Table S1). In addition, 78% of 1 pmol MO*zatxn7^AUG^* morphants that were still alive at 24 hpf (*n*  = 66) displayed severe developmental defects, including impaired head or tail differentiation or both (Fig. 3E). These phenotypes were not observed in either non-injected siblings or morphants that had received 1 pmol 5 mismatch-containing MO*zatxn7^AUG^* (mmMO*zatxn7^AUG^*) (Figure 3C and 3D), suggesting an essential requirement for zebrafish atxn7 in proper embryonic development.

To assess the specificity of the phenotypes observed in MO*zatxn7^AUG^* morphants and also test whether maternal transcripts underlie the requirement for zebrafish *atxn7* in early development, embryos of the wild-type AB strain were microinjected with a second MO targeting the donor splice site of zebrafish *atxn7* intron 4 (MO*zatxn7^SPL^*) (Figure 3A). Following microinjection of 1 pmol MO*zatxn7^SPL^*, morphants displayed high levels of embryonic lethality, with 27.6% and 61% of injected embryos dying before 10 and 24 hpf, respectively (n  = 141) (Table S1). Also, 76% of 1 pmol MO*zatxn7^SPL^* morphants that were still alive at 24 hpf (*n*  = 55) showed developmental defects similar to those seen in 1 pmol MO*zatxn7^AUG^* morphants (Figure 3F). To further confirm the specificity of MO*zatxn7^SPL^* and estimate the levels of zebrafish atxn7 depletion in the corresponding morphants, we carried out a RT-PCR analysis of zebrafish *atxn7* intron 4 splicing in 24 hpf embryos that had received 0.3, 0.6 or 1 pmol MO*zatxn7^SPL^*. RT-PCR experiments were performed using a pair of primers designed to amplify a cDNA fragment encompassing zebrafish *atxn7* exons 3 to 5 (Figure 3A). The splice-blocking activity of MO*zatxn7^SPL^* was evidenced by the dose-dependent inhibition of zebrafish *atxn7* intron 4 splicing in MO*zatxn7^SPL^* morphants (Figure 3B). We note that a nearly complete inhibition of intron 4 splicing was observed in 1 pmol MO*zatxn7^SPL^* morphants.

### Mild Zebrafish atxn7 Depletion Compromises Photoreceptor Differentiation

**Figure 4:**
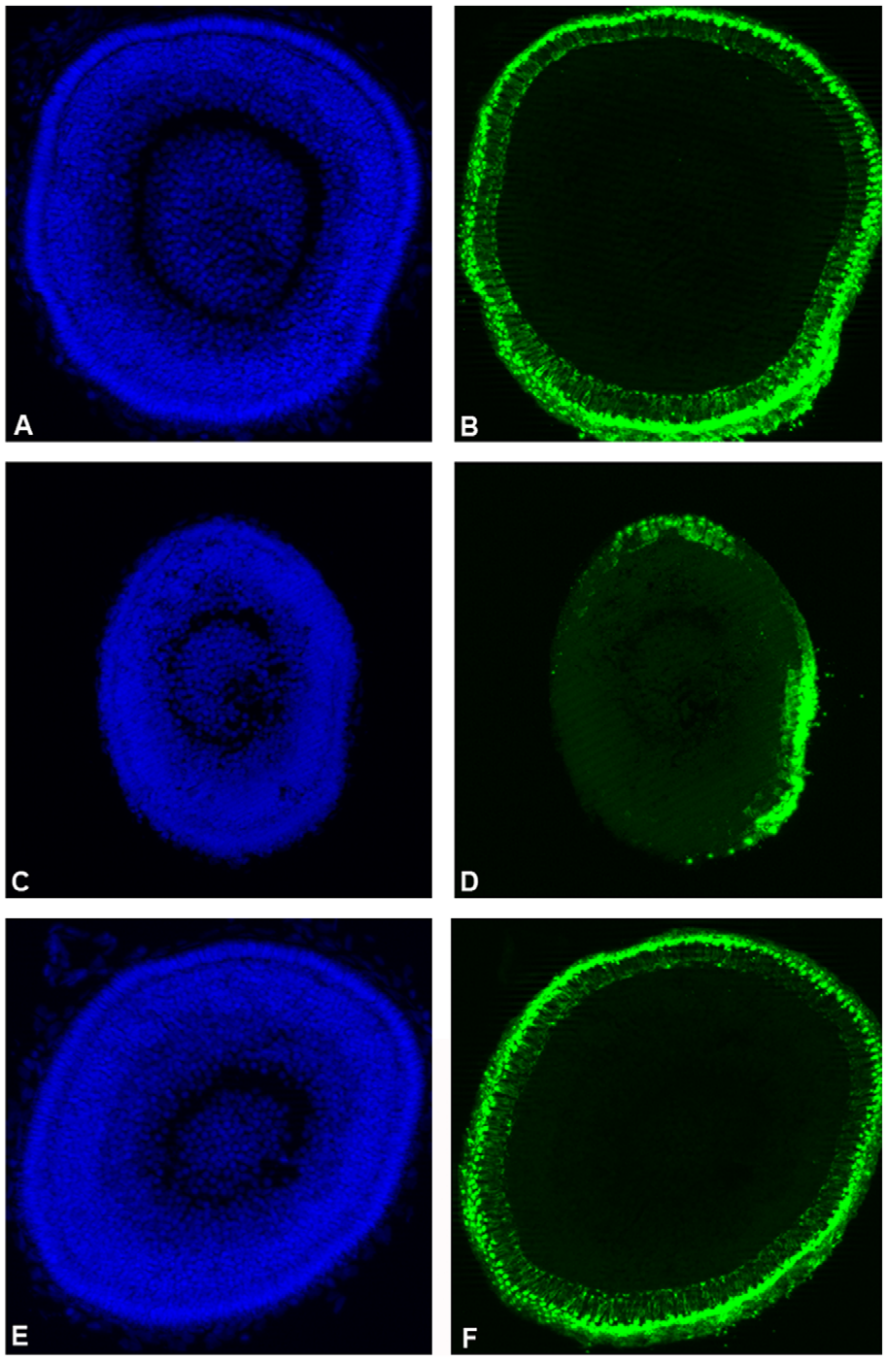
Partial zebrafish atxn7 depletion impairs photoreceptor differentiation. DAPI staining (A, C, and E) and rhodopsin immunostaining (B, D, and F) of eye cryosections of 48 hpf wild type embryo (A and B) and age-matched 0.3 pmol MO*zatxn7^SPL^* (C and D) and 1 pmol mmMO*zatxn7^AUG^* morphant embryos (E and F).

While embryos that had received 0.3 pmol MO*zatxn7^AUG^* or 0.3 pmol MO*zatxn7^SPL^* did not display obvious developmental defects nor excessive lethality (Table S1), 15% (*n*  = 113) and 12% (*n*  = 125) of these embryos showed partially depigmented retina, respectively (Figure S2). To further investigate the requirement for zebrafish atxn7 in retina differentiation, eye sections of 3 days post-fecundation (dpf) 0.3 pmol MO*zatxn7^SPL^* morphants were analysed by immunocytochemistry using an anti-rhodopsin antibody. In all the retinas analysed (*n*  = 8), whatever their pigmentation, we observed a marked disorganization of the photoreceptor layer (Figure 4C and 4D). These phenotypes were absent in both age-matched non-injected siblings (*n*  = 6) (Figure 4A and 4B) and 1 pmol mmMO*zatxn^AUG^* morphants (*n*  = 7) (Figure 4E and 4F). Importantly, rhodopsin immunostaining also revealed that 0.3 pmol MO*zatxn^SPL^* morphants displayed a marked reduction in the number of photoreceptors (Figure 4C and 4D), demonstrating an essential requirement for zebrafish atxn7 in the full differentiation of retina photoreceptors.

### Moderate Zebrafish atxn7 Depletion Specifically Impairs Purkinje and Granule Cell Differentiation

**Figure 5:**
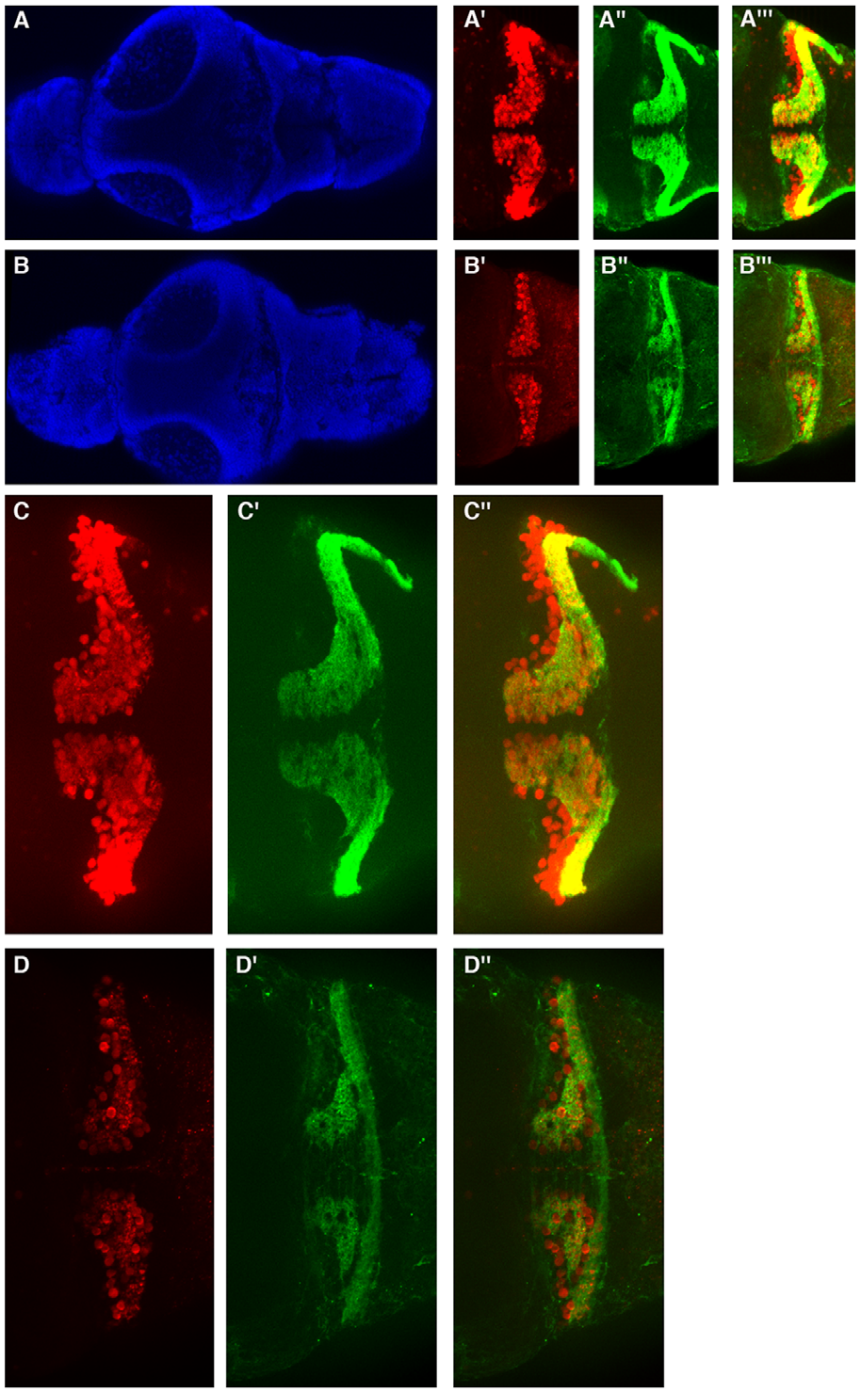
Moderate zebrafish atxn7 depletion impairs the differentiation of cerebellar neurons. Dorsal views of dissected brains from 5 dpf 1 pmol mmMO*zatxn^AUG^* (A, A’, A’’, A’’’, C, C’ and C’’) and 0.3 pmol MO*zatxn7^SPL^* morphants (B, B’, B’’, B’’’, D, D’ and D’’). DAPI staining (A and B), Pav7 immunostaining of Purkinje cells (A’, B’, C and D) and Vglut1 immunostaining of granule cells (A’’, B’’, C’ and D’). Anterior is to the left. Enlarged views of the brains showed in A’ (C), A’’ (C’), A’’’ (C’’), B’ (D), B’’ (D’), and B’’’ (D’’). Merge images of the photographs A’ and A’’ (A’’’), B’ and B’’ (B’’’), C and C’ (C’’), and D and D’ (D’’).

**Figure 6:**
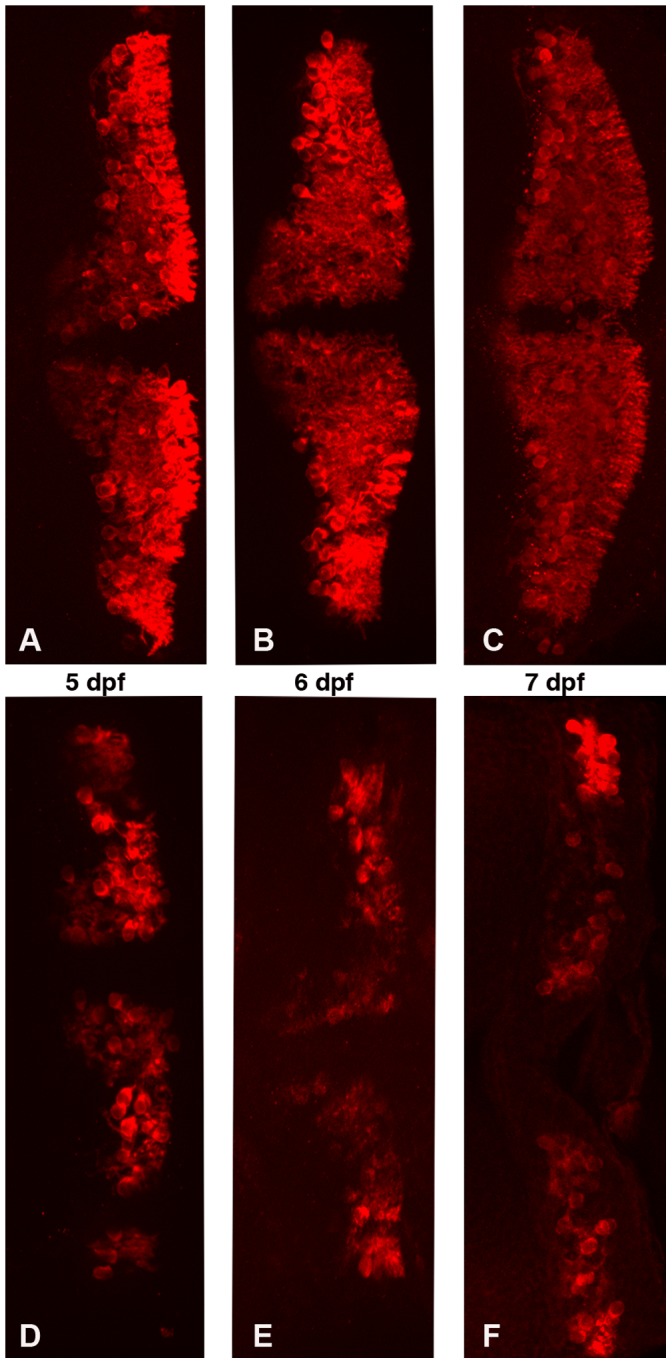
Partial zebrafish atxn7 depletion impairs the differentiation of cerebellar neurons. Dorsal views of dissected brains from 5 (A and D), 6 (B and E), and 7 dpf (C and F) 1 pmol mmMO*zatxn7^AUG^* (A, B, and C) and 0.3 pmol MO*zatxn7^SPL^* morphants (D, E, and F) immunostained with an anti-zebrin II antibody, which specifically labels Purkinje cells.

Because cerebellar neurons are particularly prone to degeneration in SCA7, we next tested whether partial depletion of zebrafish atxn7 in 0.3 pmol MO*zatxn7^SPL^* morphants induced an impaired differentiation of cerebellar neurons. First, we determined the number of Purkinje cells on consecutive serial optic sections of brains dissected from 5 dpf 0.3 pmol MO*zatxn7^SPL^* morphants by immunocytochemistry using an anti-paravalbumin-7 (Pvalb7) antibody that specifically labels these neurons [34]. In 5 dpf embryos that had received 1 pmol mmMO*zatxn^AUG^*, we detected 191+/−8 Purkinje cells (*n*  = 7) (Figure 5A’ and 5C). By contrast, the number of Pvalb7-expressing cells was very significantly reduced to 79+/−25 (*n*  = 8, *p*<0.0001) in 5 dpf embryos that had received 0.3 pmol MO*zatxn7^SPL^* (Figure 5B’ and 5D), suggesting a requirement for zebrafish atxn7 in Purkinje cell differentiation. To further investigate the physiology of Purkinje cells in 0.3 pmol MO*zatxn^SPL^* morphants, we analysed the expression of three additional proteins that have been shown to accumulate to high levels in zebrafish Purkinje cells, namely zebrin II, carbonic anhydrase 8 (Ca8) and retinoic-related orphan receptor α (RORα) [34]. In brains of 5 dpf 0.3 pmol MO*zatxn7^SPL^* morphants, we observed a marked decrease in the number of cells expressing RORα (Fig. S3B and S3B’’) compared with 5 dpf embryos that had received 1 pmol mmMO*zatxn^AUG^* (Figure S3A and S3A’’). Similarly, the number of cells expressing either zebrin II (Figure 6A and 6D) or Ca8 (not shown) was markedly reduced in 0.3 pmol MO*zatxn7^SPL^* morphants, compared with embryos that received 1 pmol mmMO*zatxn7^AUG^*. These results confirm that differentiation of Purkinje cells was severely compromised following moderate depletion of zebrafish atxn7. In addition to photoreceptor and Purkinje cell degeneration, SCA7 patients also show progressive loss of granule cells [18], [31], a defect also observed in SCA7 mouse models [35] and *in vitro* [36]. To test whether moderate zebrafish atxn7 depletion also affects the differentiation of granule cells, dissected brains of 0.3 pmol MO*zatxn7^SPL^* and 1 pmol mmMO*zatxn^AUG^* morphants were analysed by immunocytochemistry using an antibody directed against Vglut1, a vesicular glutamate transporter, which is expressed at high levels in zebrafish granule cells [34]. Our results demonstrate a marked decrease in the number of cells expressing Vglut1 in 5 dpf 0.3 pmol MO*zatxn7^SPL^* morphants (Figure 5B’’ and 5D’) compared with age-matched controls that received 1 pmol mmMO*zatxn7^AUG^* (Figure 5A’’ and 5C’), showing that granule cell differentiation also was partially compromised following moderate zebrafish atxn7 depletion.

As the reduced number of Purkinje cells observed in 5 dpf 0.3 pmol MO*zatxn7^SPL^* morphants might be caused by delayed differentiation of these neurons, we compared the number of zebrin II-expressing cells in dissected brains of 1 pmol mmMO*zatxn^AUG^* and 0.3 pmol MO*zatxn7^SPL^* morphants at 5, 6 and 7 dpf. In embryos injected with 1 pmol mmMO*zatxn^AUG^* and aged 5, 6, and 7 dpf, we observed 183+/−11 (*n*  = 5), 204+/−7 (*n*  = 6), and 244+/−13 (*n*  = 5) zebrin II-expressing cells, respectively (Figure 6A–6C). By contrast, in 0.3 pmol MO*zatxn7^SPL^* morphants, we observed a highly significant decrease in the number of Purkinje cells at 5 (90+/−18, *n*  = 5, *p*<0.0001), 6 (104+/−25, *n*  = 6, *p*<0.0001), and 7 dpf (143+/−23, *n*  = 6, *p*<0.0001) (Figure 6D–6F), strongly suggesting that partial zebrafish atxn7 depletion partly compromised, but did not slow down Purkinje cell differentiation.

We could not rule out the possibility that the reduced number of cerebellar neurons observed in 5 dpf 0.3 pmol MO*zatxn7^SPL^* morphants was caused by apoptosis of these cells soon after their differentiation. Accordingly, to assess whether moderate depletion of zebrafish atxn7 induced increased levels of brain neuron apoptosis, we performed a TUNEL assay on dissected brains of 5 dpf 1 pmol mmMO*zatxn^AUG^* (*n*  = 7) and 0.3 pmol MO*zatxn^SPL^* morphants (*n*  = 6), and age-matched DNase-treated wild-type controls (*n*  = 6). While numerous labelled cells scattered throughout the brain were detected following DNase treatment (Figure S4B), a low and roughly similar number of apoptotic cells was detected in both 1 pmol mmMO*zatxn^AUG^* (Figure S4D) and 0.3 pmol MO*zatxn^SPL^* morphants (Figure S4F), demonstrating that increased levels of apoptosis was not the cause of the reduced number of cerebellar neurons observed in 0.3 pmol MO*zatxn7^SPL^* morphants.

### [1–232] Fragment of Human atxn7 can Compensate Partial Loss of Zebrafish atxn7

**Figure 7:**
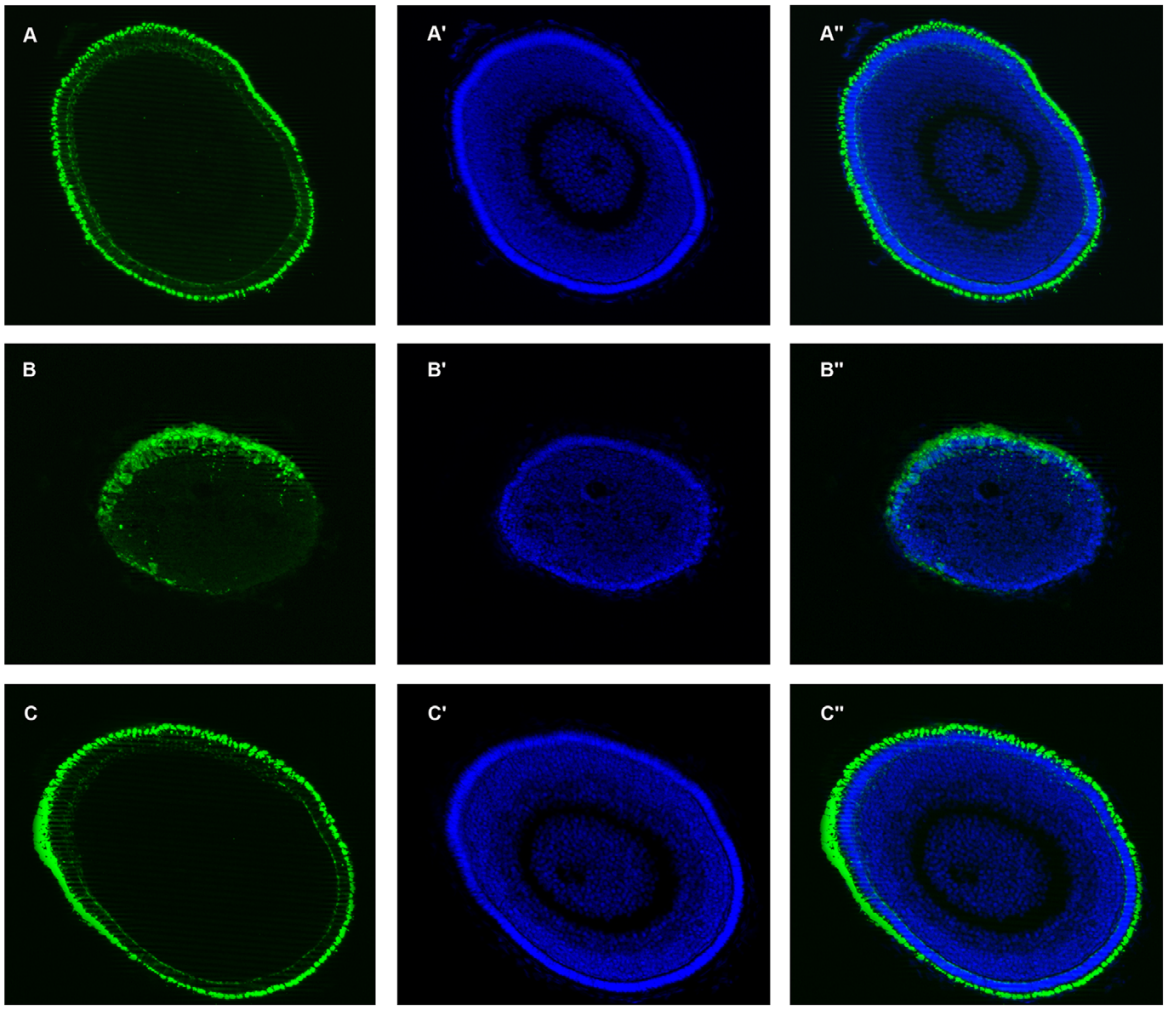
[1–232] N-terminal fragment of human atxn7 can rescue photoreceptor differentiation defect in 0.3 pmol MO*zatxn7^SPL^* morphant. Rhodopsin immunostaining (A, B, and C) and DAPI staining (A’, B’, and C’) of eye cryosections of 48 hpf 1 pmol mmMO*zatxn7^AUG^* (A, A’ and A’’) and 0.3 pmol MO*zatxn7^SPL^* morphants (B, B’ and B’’) and age matched 0.3 pmol MO*zatxn7^SPL^* morphant co-injected with 2 fmol human *atxn7* mRNA (C, C’ and C’’). Merge images of the photographs A and A’ (A’’), B and B’ (B’’), and C and C’ (C’’).

**Figure 8:**
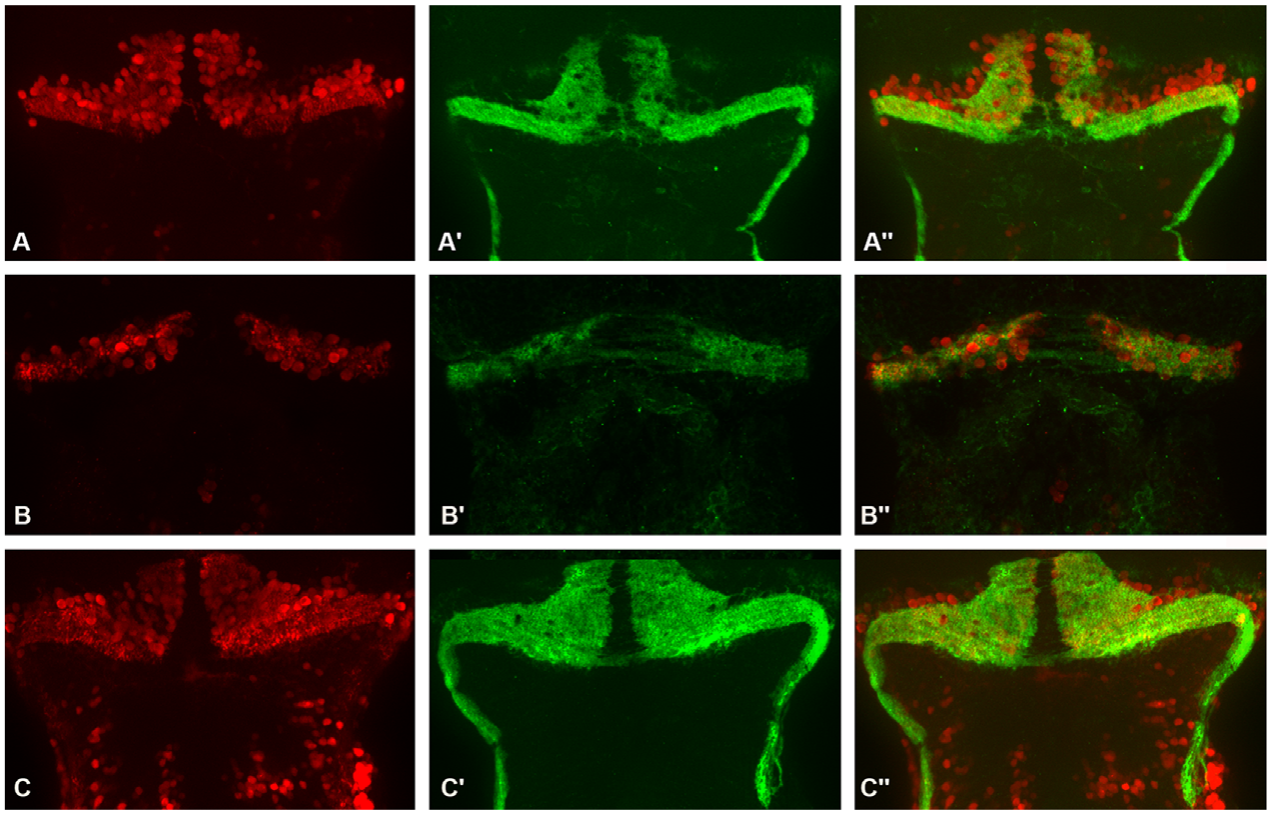
[1–232] human atxn7 fragment can rescue differentiation defects of cerebellar neurons in 0.3 pmol MO*zatxn7^SPL^* morphant. Dorsal views of dissected brains from 5 dpf 1 pmol mmMO*zatxn^AUG^* (A, A’ and A’’) and 0.3 pmol MO*zatxn7^SPL^* morphants (B, B’ and B’’) and age-matched 0.3 pmol MO*zatxn7^SPL^* morphant co-injected with human *atxn7* mRNA (2 fmol) (C, C’ and C’’). Pav7 immunostaining of Purkinje cells (A, B and C) and Vglut1 immunostaining of granule cells (A’, B’ and C’). Anterior is to the left. Merge images of the photographs A and A’ (A’’), B and B’ (B’’), and C and C’ (C’’).

To further confirm that loss of both photoreceptors and cerebellar neurons observed in 0.3 pmol MO*zatxn7^SP^* ^L^ morphants (Figure 4 and Figure 5) was caused by partial depletion of zebrafish atxn7, and also to test whether human atxn7 could compensate for loss of function of the zebrafish protein, wild-type embryos were injected with a mixture comprising 0.3 pmol MO*zatxn7^SPL^* and 2 fmol of *in vitro* transcribed human *atxn7* mRNA encoding truncated human atxn7 (ATXN7T: amino acids 1–232), which has successfully been used to model SCA7 *in vivo* in *Drosophila* [37] and is close to the shortest human atxn7 fragment found in human brain or transgenic SCA7 mice [38]. In all the embryos that received 0.3 pmol MO*zatxn7^SPL^* and 2 fmol truncated human *atxn7* mRNA encoding [1–232] N-Terminal fragment of the protein (*n*  = 78), retina pigmentation was similar to that observed in non-injected siblings or 1 pmol mmMO*zatxn7^AUG^* morphants (not shown). Also, in all the retinas analysed (*n*  = 7), rhodopsin immunostaining of eye sections revealed that differentiation of photoreceptors was similar in 0.3 pmol MO*zatxn7^SPL^* morphants that also received 2 fmol truncated human *atxn7* mRNA (Figure 7C–7C’’), compared with non-injected embryos (data not shown) or 1 pmol mmMO*zatxn^AUG^* morphants (Figure 7A–7A’’). Next, we investigated whether [1–232] fragment of human atxn7 was also able to rescue cerebellar neuron differentiation defects observed in 0.3 pmol MO*zatxn7^SPL^* morphants (Figure 5). In the dissected brains of morphants that received 0.3 pmol MO*zatxn7^SPL^* and 2 fmol truncated human *atxn7* RNA (*n*  = 6), we detected 186+/−12 (*n*  = 6) Purkinje cells as revealed by Pvalb7 immunostaining (Figure 8C and 8C’’), a number similar to that observed in 1 pmol mmMO*zatxn^AUG^* morphants (191+/−8, *n*  = 7, *p*  = 0.4) (Figure 8A and 8A’’). Similarly, Vglut1 immunostaining demonstrated that the number of granule cells seen in dissected brains of 0.3 pmol MO*zatxn7^SPL^* morphants that also received 2 fmol truncated human *atxn7* RNA (Fig. 8C’ and 8C’’) was similar to that observed in 1 pmol mmMO*zatxn7^AUG^* morphants (Fig. 8A’ and 8A’’). Taken together, these data demonstrate that [1–232] N-terminal fragment of human atxn7 was able to fully rescue cerebellar neuron and photoreceptor differentiation defects caused by partial depletion of the zebrafish protein.

### Partial Zebrafish atxn7 Depletion does not Affect Overall Brain, Spinal Cord and Muscle Development

To determine whether the differentiation of other brain neurons and/or glial cells were also compromised following mild zebrafish atxn7 depletion, we analysed the accumulation pattern of both the glial acidic fibrillary protein (GFAP), a protein accumulated at high levels in all glial cells [39], and HuC, a pan-neuronal protein, which is expressed in all brain neurons [40], in dissected brains of 5 dpf 0.3 pmol MO*zatxn7^SPL^* morphants (*n*  = 5) and age-matched control embryos (*n*  = 6). No differences in either the size and organization of brain regions or the accumulation pattern of the two proteins could be detected between 5 dpf wild-type controls (Figure S5A-S5C) and age-matched 0.3 pmol MO*zatxn7^SPL^* morphants (Figure S5D-S5F), suggesting that overall brain organization was not impaired following moderate zebrafish atxn7 loss of function.

We also analysed whether partial depletion of zebrafish atxn7 caused defects in spinal cord development, motor neuron differentiation or body muscle structure and/or organization. We first made use of the Tg[NBT:MAPT-GFP]zc1 transgenic line to visualize the spinal cord and motor neuron axons [41]. Following injection of 0.3 pmol MO*zatxn7^SPL^* in embryos of the Tg[NBT:MAPT-GFP]zc1 line, we observed that both spinal cord anatomy and motor neuron axon arborisation were fully similar in 0.3 pmol MO*zatxn7^SPL^* morphants (*n*  = 6) (Figure S6B) compared with non-injected embryos (*n*  = 5) (Figure S6A). Next, we examined trunk muscle organization in 48 hpf 0.3 pmol MO*zatxn7^SPL^* morphants (*n*  = 6) and age-matched 1 pmol mmMO*zatxn7^AUG^* controls (*n*  = 5) using labelling with rhodamine-coupled phalloidin, an F-actin binding molecule that allows visualization of muscle fibres. We were unable to detect any differences between the morphology of trunk muscle of 48 hpf 0.3 pmol MO*zatxn7^SPL^* morphants (Figure S7B) and that observed in 1 pmol mmMO*zatxn7^AUG^* morphants at the same stage (Figure S7A). Taken together, these data show that differentiation of body muscles, spinal cord and motor neurons were not impaired following mild depletion of zebrafish atxn7 in embryos.

## Discussion

The potential prevalence of mutations that lead to both loss and gain of function in human neurological disease (as shown by the phenotypes of presenilin^−/−^ mice) [42], [43] underscores the importance of understanding endogenous functions of causative genes through careful analysis of loss-of-function models, which may uncover critical pathways leading to pathogenesis. Here, we performed a functional analysis of zebrafish atxn7 in the vertebrate teleost fish *D. rerio* (zebrafish) and investigated the phenotypes caused by various levels of protein depletion. We established a specific requirement for atxn7 in proper differentiation of the three main neuronal populations that are vulnerable in SCA7, i.e. photoreceptors, and cerebellar Purkinje and granule cells. Although the loss of differentiated neurons observed in SCA7 is clearly distinct from the neuronal differentiation defect seen in MO*zatxn7* morphants, the similarity in the neuronal populations affected in the two processes is highly intriguing and suggest that reduced, or altered atxn7 function, in SCA7 plays a role in the pathophysiology of the disease. Moreover, these data are in good agreement with recent *in vitro* results suggesting that atxn7 plays role in Purkinje cell development and differentiation (Latouche et al., unpublished data). This loss could be the result of either dominant-negative activity of expanded atxn7 as discussed below or partial loss of function caused by heterozygocity of the non-expanded allele combined with progressive trapping of the wild-type protein in neuronal intranuclear inclusions (NII) [44], [45], or both. The dominant mode of inheritance of all polyQ-expansion diseases together with the deleterious effect of isolated polyQ peptides *in vitro* [46] and *in vivo* [46]–[49], led to the suggestion that pathologically expanded polyQ tract endowed the causative proteins with either a toxic gain of function or a dominant negative activity detrimental to life-long living post-mitotic neurons. However, a body of work also suggests that protein loss of function may also play a role in the disease phenotype in several polyQ disorders, such as SCA6 [50], [51], or HD [52]. In the case of SCA7, while our data suggest that altered atxn7 function plays a role in the disease phenotype, this hypothesis was challenged by the observation that transgenic mice homozygous for an expanded *atxn7^266Q^* allele, SCA7^266Q/266Q^ mice, but not hemizygous SCA7^266Q/−^ mice, displayed a worsened phenotype compared with SCA7^266Q/+^ animals [33]. However, it is important to keep in mind that such huge expansions in SCA7 (>200 residues), which have been described in only very few cases, induce pathologies that can no longer be classified as SCA, the disease, manifest from the first weeks post-pregnancy onward, affecting several non-neuronal tissues, such as the heart, kidneys and liver, and causing early lethality during the very first years of life [53]–[56]. In this context, whether partial depletion of the non-expanded protein participated in the pathophysiology of SCA7 remains an open question; further studies are required to evaluate the importance of altered atxn7 function in the disease process.

In the case of zebrafish *atxn7*, in close agreement with the results observed in mammals [16], [31], [32], [57], the gene is expressed throughout embryonic development and in several neuronal populations, including the cerebellum, spinal cord, optic tectum and telencephalon. However, we observed that only the differentiation of photoreceptors and cerebellar neurons was impaired following moderate zebrafish atxn7 depletion. The fact that differentiation of photoreceptors was compromised following partial loss-of-function of the zebrafish protein was consistent with data from the analysis of several SCA7 mouse models. Indeed, it has been shown that selective vulnerability of photoreceptors in SCA7 is related to interference of the mutant protein with CRX [27], [29], [30], a homeobox transcription factor crucial for photoreceptors differentiation through transcriptional regulation of photoreceptor-specific genes [58]–[61] and a direct partner of atxn7 [27]. By analogy, our results suggest that atxn7-SAGA might also be a partner of a transcription factor crucial for either differentiation and maintenance of cerebellar neurons or proper expression of another factor essential for cerebellar neuron differentiation and physiology. RORα, a transcription factor belonging to the family of retinoid-related orphan nuclear-receptors and crucial for proper differentiation of Purkinje cells [62], [63], appears as a good candidate. Indeed, *rorα* was also down-regulated in a knock-in transgenic mouse model of SCA1 (*atxn1^82Q/82Q^*), but not in *atxn1^−/−^* mice [64], [65] and partial loss of function of the *rorα* gene enhanced the pathogenicity of atxn1^82Q^ [64]. All these observations raised the question of whether partial atxn7/SAGA-mediated RORα loss of function also underlay Purkinje cell loss in *MOzatxn7* morphant embryos and also possibly in SCA7 patients. The finding that down-regulation of *rorα* or partial loss of RORα activity plays a role in the pathophysiology of SCA7 would be an important step toward the development of new therapeutic strategies. Further work is now required to evaluate the role of RORα in cerebellar neuron degeneration in SCA7.

Blast analysis identified 4 *atxn7* paralogs in zebrafish, *zatxn7*, *zatxn7l2a* and *l2b*, and *zatxn7l3*, which are the orthologs of the human *atxn7*, *atxn7L2* and *atxn7L3* genes, respectively. Although our data show a specific requirement for zebrafish atxn7 in embryonic development and differentiation of photoreceptors and cerebellar neurons, the identification of atxn7L2 and atxn7L3 as components of the SAGA complex [66], [67], suggests a possible functional redundancy of these proteins.

Finally, the ability of human atxn7 to compensate the loss of the zebrafish protein in *D. rerio* embryos emphasizes the conservation of the function of this protein during evolution and thus, the interest of this fish as model to test therapeutic hypotheses.

## Materials and Methods

### Animals

Zebrafish were maintained at 28°C in a standard zebrafish facility (Aquatic Habitats, Apopka, FL. U.S.A.) as described in Westerfield [68]. Developmental stages were determined as hours post-fertilization (hpf) as described by Kimmel et al. [69]. Wild-type embryos were from the *AB* and *TL* strains. For *in situ* hybridization and immunohistochemistry, embryos were treated with 0.005% phenylthiourea from 20 hpf onward to prevent pigmentation.

### Validation of the Structure and Sequences of the Zebrafish *atxn7* Gene

Zebrafish *atxn7* sequences were found in Ensembl (ENSDARG00000074804). To confirm the *in silico* data, the coding sequence of the gene was amplified from reverse-transcribed adult zebrafish cDNA, using overlapping zebrafish *atxn7*-specific primer sets (available from the authors on request) and directly sequenced using the BigDye technology (Applied Biosystem) in an ABI3730 automated sequencer. Experimental sequences were subsequently aligned with *in silico* predictions using Autoassembler (Applied Biosystems), and the consensus sequence was then analysed by UCSC Blat (http://genom.ucsc.edu/) to find the exon-intron boundaries and splice site locations. The zebrafish atxn7 protein sequence was then aligned with human and mouse counterparts using Align (http://www.ebi.ac.uk/Tools/emboss/align/) to determine domain conservation.

### *In situ* Hybridization

The *in situ* detection of zebrafish *atxn7* transcripts on dissected brains was carried out as described in Ayari et al. [70]. *In situ* hybridization on whole-mount embryos was performed principally as described in Yanicostas et al. [71]. The *in situ* hybridization of adult brain sections was done according to Bae et al. [34].

### Immunohistochemistry

Immunohistochemistry on either dissected brains or brain sections was carried out as previously described in Ayari et al., [70]. Primary antibodies anti-paravalbumin7 (anti-Pavlb7, 1/1000, mouse ascites), anti-carbonic 8 (anti-Ca8, 1/100, mouse hybridoma supernatant), anti-vesicular1 glutamate transporter (anti-Vglut1, 1/1000, rabbit purified antibody), anti-zebrin II (1/200, hybridoma supernatant) [72] were used as described in Bae et al. [34]. The rabbit anti-GFAP (DAKO, used at 1∶1000 dilution), and human anti-HuC antibodies (kindly provided to us by Jean-Yves Delattre, used at 1∶4000 dilution). Eye cryosections were incubated with anti-rhodopsin rho4D2 antibodies (generous gift of Drs Serge Picaud and David Hicks, used at 1∶500 dilution) [73]. Primary antibodies were detected using fluorescently labelled secondary antibodies; Alexa 488-coupled goat-anti-rabbit antibodies (Molecular Probes, used at 1∶250 dilution), or with the corresponding biotinylated anti-human antibodies (MP Biomedicals Cappel, used at 1∶500 dilution) diluted in blocking solution. Secondary biotinylated antibodies were visualized by incubation with Alexa 555-coupled streptavidin (Molecular Probes, used at 1∶700 dilution) diluted in PBS. Following immunostaining, dissected brains were mounted on 1% agarose in PBS and photographed using an epifluorescent AXIO imager Z.1 microscope (Zeiss) equipped with an ApoTome system (Zeiss).

### Morpholino-mediated Gene Inactivation

All morpholinos (MO) were designed by and obtained from GeneTools. To inactivate the translation of zebrafish *atxn7* RNAs, we designed a morpholino-oligonucleotide, MO*zatxn7^AUG^* (5′-CGTCATCATCGGCCCTTTCCGACAT-3′), which is complementary to the sequence flanking the translation initiating codon of the messenger RNA (underlined). We also designed a second morpholino, MO*zatxn7^SPL^* (5′-ATCAAAACACACATACACACCTCTC-3′) that targets the donor site of the fourth intron of zebrafish *atxn7* mRNA to impair proper splicing of this intron. As a control, we first designed a morpholino oligonucleotide derived from MO*zatxn7^AUG^* but comprising five mismatching bases (lower case letters), mmMO*zatxn7^AUG^,* (5′-CcTgATCATcGcCCCTTTgCGACAT-3′). We also used a non-specific morpholino oligonucleotide, MO control (5′-CCTCTTACCTCAGTTACAATTTATA-3′). For morpholino-mediated transcript inactivation experiments, 2 nl of 0.15, 0.3 or 0.5 mM solutions, and corresponding to 0.3, 0.6, and 1 pmol of the different morpholinos, respectively, were injected in 1- to 2-cell stage embryos using standard protocols.

### RT-PCR Analysis of MO*zatxn^SPL^* Splice-blocking Activity

To check the efficiency of MO*zatxn7^SPL^*-mediated splice inhibition, a reverse transcription polymerase chain reaction (RT-PCR) was performed using RNAs, which were extracted from embryos that had received 0.3, 0.6, and 1 pmol MO*zatxn7^SPL^.* RNAs were isolated using the RNeasy mini Kit (Qiagen, Germantown, MD, USA) and then reverse transcribed as cDNA using the SuperScript II Reverse Transcriptase (Invitrogen, Carlsbad, CA) according to the manufacturer’s instructions. A cDNA fragment of zebrafish *atxn7* encompassing exons 3 to 5 was PCR amplified using the *zatxn7*-forw (5′-GGCCTTCCAAGCACATTAC-3′) and *zatxn7*-rev (5′-GTCATATCCATAACCCCAC-3′) primers.

### Phenotypic Rescue

For repscue experiments, 2 nl of a mix containing MO*zatxn7^SPL^* (0,15 mM) and human *atxn7* mRNA (1 µM), was injected into embryos at the one- to two-cell stage according to standard protocols, and the phenotypes were analysed at the indicated stages.

### TUNEL Assay

For the detection of apoptotic cells, terminal deoxynucleotidyl transferase dUTP nick end labelling (TUNEL) assay was performed on dissected brains according to Yabu et al. [74].

### Phalloidin-rhodamine Staining

Trunk muscles were visualized by phalloidin-rhodamine staining of F-actin. Briefly, 48 hpf embryos were anesthetized in tricaine, and fixed by an o/n incubation in 4% PFA in PBST (PBS, 0.1% Triton X-100) at 4°C, followed by three washes in PBST. Embryos were then incubated for 30 minutes in phalloidin-rhodamine (at 1/100) dissolved in PBS, washed three times in PBST and mounted in 1% low-melting agarose and imaged using a fluorescent microscope equipped with an ApoTome system (Zeiss).

### Ethics Statement

All procedures involving animal handling in this study complied with the guidelines of the French Animal Ethics Committee and was approved by the same committee under the ethics statement: 2012-15/676-0069.

## Supporting Information

Figure S1 **Identification and sequence of the zebrafish** ***atxn7*** **gene.** Molecular phylogeny of the human (*hatxn7* or ENSG00000163635; *hatxn7L1* or ENSG00000146776; *hatxn7L2* or ENSG00000162650; *hatxn7L3* or ENSG00000087152; and *hatxn7L3B* or ENSG00000253719) and zebrafish (*zatxn7* or ENSDARG00000074804; *zatxn7l2a* or ENSDARG00000055300; *zatxn7l2b* or ENSDARG00000056268; and *zatxn7l3* or ENSDARG00000029331) *atxn7* paralogs (A). RT-PCR analysis of the transcription of the *zatxn7l2a*, *zatxn7l2b* and *zatxn7l3* genes in zebrafish embryos aged 24, 48 and 72 hpf (B). Sequence alignment of the human (H.s., ENSG00000163635), mouse (M.m., ENSMUSG00000021738), and zebrafish (D.r., ENSDARG00000074804) atxn7 protein sequences (C). All the sequences were obtained from the Ensembl data base (http://www.ensembl.org). Molecular phylogeny was determined using ClustalW2 (http://www.ebi.ac.uk/Tools/msa/clustalw2/). Peptidic sequences were aligned using Align (http://www.ebi.ac.uk/Tools/msa/clustalw2/). Colour code for amino acids: identical amino acids, red; similar amino acids, blue. Abbreviations: *Homo sapiens*, H.s.; *Mus musculus*, M.m.; *Danio rerio*, D.r.
(DOCX)

Figure S2 **Mild zebrafish atxn7 depletion impairs retina differentiation.** 48 hpf 1 pmol mmMO*zatxn7^AUG^* (A) and 0.3 pmol MO*zatxn7^AUG^* morphant embryos (B). Eye of a 48 hpf 1 pmol mmMO*zatxn7^AUG^* morphant (C) and partially depigmented retinas of 48 hpf 0.3 pmol MO*zatxn7^AUG^* (D) and 0.3 pmol MO*zatxn7^SPL^* (E and F) morphant embryos.
(TIF)

Figure S3 **Partial zebrafish atxn7 depletion impairs Purkinje cell differentiation.** Frontal sections of dissected brains of 5 dpf 1 pmol mmMO*zatxn7^AUG^* (A-A’’) and 0.3 pmol MO*zatxn7^SPL^* morphant embryos (B-B’’). RORα immunostaining of Purkinje cells (A and B) and DAPI staining (A’ and B’). Merge images of the photographs A and A’ (A’’) and B and B’ (B’’).
(TIF)

Figure S4 **Moderate zebrafish atxn7 depletion does not induce cerebellar neuron apoptosis.** Dorsal views of dissected brains from DNase-treated non-injected control (A and B) and 1 pmol mmMO*zatxn7^AUG^* (C and D) and 0.3 pmol MO*zatxn7^SPL^* morphant embryos (E and F). Anterior is to the left. DAPI staining (A, C and E) and TUNEL labelling of apoptotic cells (B, D and F). Abbreviations: TeO, tectum optic; Cer, cerebellum.
(TIF)

Figure S5 **Mild zebrafish atxn7 depletion does not impair overall brain organization.** Dorsal view of dissected brains from 5 dpf 1 pmol mmMO*zatxn7^AUG^* (A-C) and 0.3 pmol MO*zatxn7^SPL^* morphant embryos (D-F). Anterior is to the left. GFAP immunostaining of glial cells (A and D) and HuC immunostaining of neuronal cells (B and E). Merge images of the photographs A and B (C) and D and E (F).
(TIF)

Figure S6 **Partial zebrafish atxn7 depletion does not impair spinal cord differentiation.** Lateral views of 48 hpf Tg[NBT:MAPT-GFP]zc1 transgenic embryos following injection of 1 pmol mmMO*zatxn7^AUG^* (A) and 0.3 pmol MO*zatxn7^SPL^* (B). Anterior is to the left.
(TIF)

Figure S7 **Moderate zebrafish atxn7 depletion does not impair the differentiation of trunk muscles.** Lateral views of 48 hpf 1 pmol mmMO*zatxn7^AUG^* (A) and 0.3 pmol MO*zatxn7^SPL^* morphant embryos (B) following rhodamine-coupled phalloidin labelling of muscle F-actin. Anterior is to the left.
(TIF)

Table S1 **Phenotypes of zebrafish** ***atxn7*** **knockdown embryos.**
(DOCX)
