# Supplementary material for: Requirement for Zebrafish Ataxin-7 in Differentiation of Photoreceptors and Cerebellar Neurons
Source: PLoS One. 2012 Nov 30;7(11):e50705. doi: 10.1371/journal.pone.0050705 (PMC3511343; doi:10.1371/journal.pone.0050705)
Supplement: Figure S1 — Identification and sequence of the zebrafish atxn7 gene. Molecular phylogeny of the human (hatxn7 or ENSG00000163635; hatxn7L1 or ENSG00000146776; hatxn7L2 or ENSG00000162650; hatxn7L3 or ENSG00000087152; and hatxn7L3B or ENSG00000253719) and zebrafish (zatxn7 or ENSDARG00000074804; zatxn7l2a or ENSDARG00000055300; zatxn7l2b or ENSDARG00000056268; and zatxn7l3 or ENSDARG00000029331) atxn7 paralogs (A). RT-PCR analysis of the transcription of the zatxn7l2a, zatxn7l2b and zatxn7l3 genes in zebrafish embryos aged 24, 48 and 72 hpf (B). Sequence alignment of the human (H.s., ENSG00000163635), mouse (M.m., ENSMUSG00000021738), and zebrafish (D.r., ENSDARG00000074804) atxn7 protein sequences (C). All the sequences were obtained from the Ensembl data base (http://www.ensembl.org). Molecular phylogeny was determined using ClustalW2 (http://www.ebi.ac.uk/Tools/msa/clustalw2/). Peptidic sequences were aligned using Align (http://www.ebi.ac.uk/Tools/msa/clustalw2/). Colour code for amino acids: identical amino acids, red; similar amino acids, blue. Abbreviations: Homo sapiens, H.s.; Mus musculus, M.m.; Danio rerio, D.r. (DOCX) [file pone.0050705.s001.docx]

**
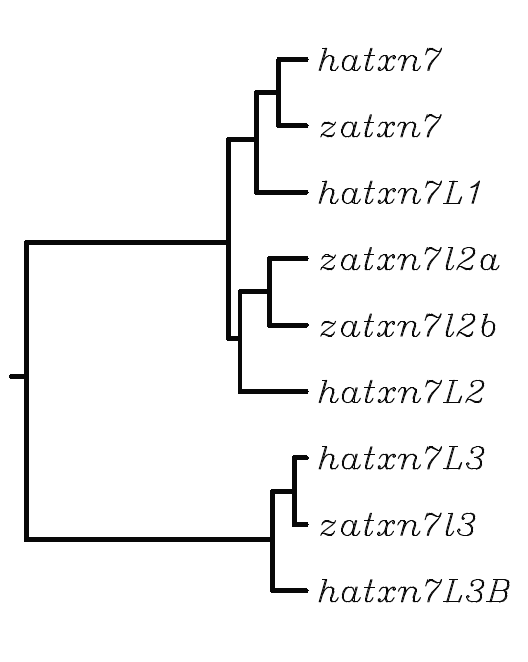
A B**


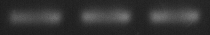

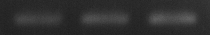

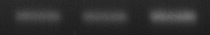

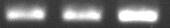


*zatxn7l2a*

*zatxn7l2b*

*zatxn7l3*

*ß-actin*

24 hpf 48 hpf 72hpf

**C**

H.s. MSERAADDVRGEPRRAAAAAGGAAAAAARQQQQQQQQQQPPPPQPQRQQHPPPPPRRTRPEDGGPGAASTSAAAMATVGERRPLPSPEVMLGQSWNLWVEASKL 104

M.m. MSERAADDVRGEPRRA-----AGGAAAARQ-QQQQPQ----PLQPQRQ-HPPLRRPRA—-EDGGTGDTTTSAAAMATVGERRPLPSPEAMLGQSWNLWVEASKL 91

D.r. MSERADDDVRGEQQQQQRR-------AARQ-LKQQPHF-------------------------QRGEAST---AMATVAERRSLPSPEIMLGQPWSSWVDAVKL 68

H.s. PGKDGTELDESFKEFGKNREVMGLCREDMPIFGFCPAHDDFYLVVCNDCNQVVKPQAFQSHYERRHSSSSKPPLAVPPTSVFSFFPSLSKSKGGSASGSNRSSS 208

M.m. PGKDGTELDESFKEFGKNREVMGLCREDMPIFGLCPAHDDFYLVVCNDCNQVVKPQAFQSHYERRHSSSSKPALAVPHTSVFSLLPSLSKSKGSGAGGSSRPPS 195

D.r. YGSDGTELEESLKECGKNREAMRLCRDDMPIFGQCPAQDDFYLVMCSHCSQVVKPQAFQAHYERRHSSSSKPPSSYASSPAFAN—-SRSKGSLGGSGSTLARPS 170

H.s. GGVLSASSSS-SKLLKSPKEKLQLRGNTRPMHPIQQSRVPHGRIMTPSVKVEKIHPKMD-----------GTLLKSAVGPTCPATVSSLVKPGLNCPSIPK-PT 302

M.m. GGVLCASSS--SKLLRLPKEKLPLRGNMKPMHPVQQIKVPHGRVMTPSVKVEKMHPKMD-----------GTLLKSTVGPACPATMSSAVKPGLNCPSIPK-PT 288

D.r. ----SANSSVPTKIFKSTKEKLPHR—--KPHFPF---RVPSEESQIPTVKVEKVHLKVESSAKLPHVPASSSTFSSS-SSSSNTVSTSPLKSGLNFPSIPKAPQ 265

H.s. LPSPGQILNGKGLPAPPTLEKKPEDNSNNRKFLNKRLSEREFDPDIHCGVIDLDTKKPCTRSLTCKTHSLTQRRAVQGRRKRFD-LLAEHKNKTREKELIRHPD 399

M.m. LPSPGQILNGKGLPAMPTLEKKSEDSSNNRKFLNKRLSEREFDPDIHCGVIDLDTKKPCTRSLTCKTHSLTQRRAVQGRRKRFD-LLAEHKNKAREKELIRH-D 384

D.r. L-APGQIPNGKGHQS--SQDKKQ-DNASSRRPLYKRQ-EREFNPDIHCGVMDMTARKPCTRSLTCKSHSISQRRAVLGRRQRFDTLLAEHKSKTRDKELQFRLD 359

H.s. SQQPPQPLRDPHPAPPRTSQEPHQNPHG-VIPSESKPFVASKPKPHTPSLPRPPGCPAQQGGSAPIDPPPVHESPHPPLPATEPASRLSSEEGEGDDKEESVEK 505

M.m. SQQVPHPLRDPHPTPPRTPQEPQLP-------AESKPFLASKPKPQTPSLPRPPGCPAQQGGSTPIDPPPGQESPHPPLPATEPASRLSSEEGEGDDREESVEK 484

D.r. PSHPAPPLRDPHPPPSRLSQDPHPVPHGNGTADATKPLPPNKPKPHNSGLPRINSSNSHSGGD-PAMAHDQAHHSHPAPVGPET-SRVSSDEGENEDREEATEK 464

H.s. LDCHYSGHHPAQP-SFCTFGSRQIGRGYYVFDSRWNRLRCALNLMVEKHLNAQLWKKIPPVPS-TTS-PISTRIPHRTNSVPTSQCGVSYLAAATVSTSPVLLS 606

M.m. LDCHYSGRHP-QPASFCTFGSRQIGRGYYVFDSRWNRLRCALNLMVEKHLNAQLWKKIPPVPC-TTS-PVSARVPHRTNSVPTSQGGISYLAATTVSAPPVLLS 585

D.r. LDCHYSGYHP-RPAAYCTFGSRLYGRGCFAFDRRWDQVRCALTTMLDKHVNSQMWKKIPLALENSTTAP-SA--SHRTSTNSHSSSS-SSVSSGFLG-----LS 557

H.s. STCISPNSKSVPAHGTTLNAQ--PAASGAMDPVCSMQSRQVSSSSSSPSTPSG-LSSVPSSPMSRKPQKLKSSKSLRPKE-SSGNSTNC----------QNASS 696

M.m. STCISPNSKSVPAHGTTLNAQ--PAGSGAMDPVCSVQSRQVSASSSPPSTPSG-LSSVPSSPLSRKPQKWKPSKSIRPKE-SSALSTNC----------HNASS 675

D.r. SPP-PYDSKPVLSYGTTLNARSSPQASATEQPAYSGSSRQVSASS—-PQMPSAHSSSLPSLGSNRTPKSRSGTKSFRAREPSSSLSNSIIKGSTNSSVSVSSSA 659

H.s. STSGGSGKKRKNSSPLLVHSS-SSSSSSSSSSHSMESFRKNCVAHSG-----------------PPYPS-TVTSSHS----IGLNNCVTNKANAVNVRHDQSGR 777

M.m. STSGGSGKKRKNSSPLLVPSSSSSSSSSSSSSHSVNSFRKNCVAHSG-----------------TPYLSVTAPSSHS----IGLNNCVTNKTHSS-LRHEQAGR 757

D.r. SSSLSAGKKRKTSSLLTSHSTYTSESSSS--------FKKNCTINSGSVGSAYHSSLASTPSSTSSSSSTTS-SSHSGVYSVGVN-CTPGRANSLSLKQESTGR 753

. H.s. GPPTGSPAESIKRMSVMVNSSDSTLSLGPFI-HQSNELPVNSHGSFSHSHTPLDKLIGKKRKCSPSSSSINNSS------------SKPTKVAKVPAVNNVHMK 868

M.m. GPAGVSSAEPIKRMSVMVNSSDSTLSLGPFITHQASELPVNP-----HSH-PLDKLIGKKRKCSPGSSTVGNSG------------SKPTKVAKLPAMNNVHMK 843

D.r. GPPSGSPAESIKRMSVVMNSSDSTLSLGPFV-HQSSD------------HHTDARLEAKRRKGSPGSSSL-NSTASGTGVGGGQGPGRP-KMAKSPSINNIHSK 842

H.s. HTGTIPGAQGLMNSSLLHQPKARP 892

M.m. HTGNISGAQGLTNNSLLHQPKARP 867

D.r. HARSIPGPPGLPNNSLIHQPKARP 866
