# Supplementary figures and images for: Requirement for Zebrafish Ataxin-7 in Differentiation of Photoreceptors and Cerebellar Neurons
Source: PLoS One. 2012 Nov 30;7(11):e50705. doi: 10.1371/journal.pone.0050705 (PMC3511343; doi:10.1371/journal.pone.0050705)

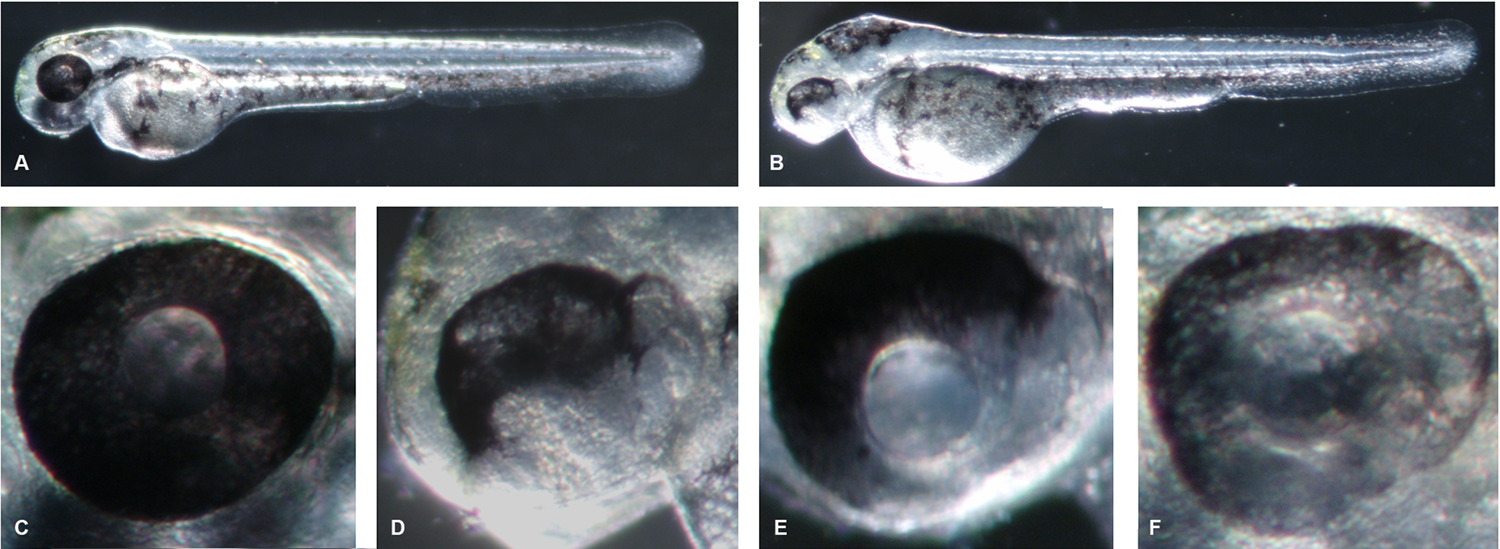

Supplement: Figure S2 — Mild zebrafish atxn7 depletion impairs retina differentiation. 48 hpf 1 pmol mmMOzatxn7AUG (A) and 0.3 pmol MOzatxn7AUG morphant embryos (B). Eye of a 48 hpf 1 pmol mmMOzatxn7AUG morphant (C) and partially depigmented retinas of 48 hpf 0.3 pmol MOzatxn7AUG (D) and 0.3 pmol MOzatxn7SPL (E and F) morphant embryos. (TIF) [file pone.0050705.s002.tif]

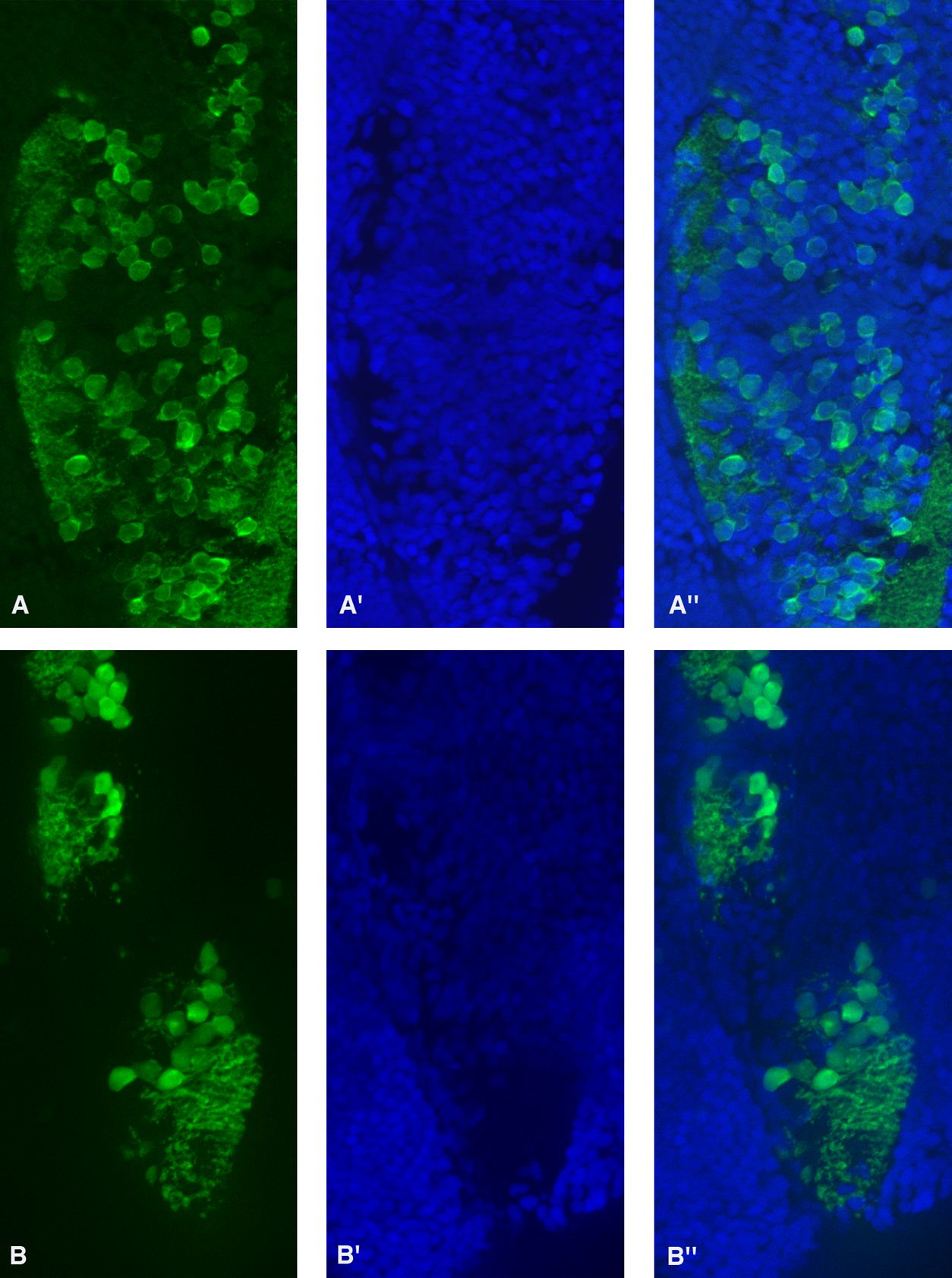

Supplement: Figure S3 — Partial zebrafish atxn7 depletion impairs Purkinje cell differentiation. Frontal sections of dissected brains of 5 dpf 1 pmol mmMOzatxn7AUG (A-A’’) and 0.3 pmol MOzatxn7SPL morphant embryos (B-B’’). RORα immunostaining of Purkinje cells (A and B) and DAPI staining (A’ and B’). Merge images of the photographs A and A’ (A’’) and B and B’ (B’’). (TIF) [file pone.0050705.s003.tif]

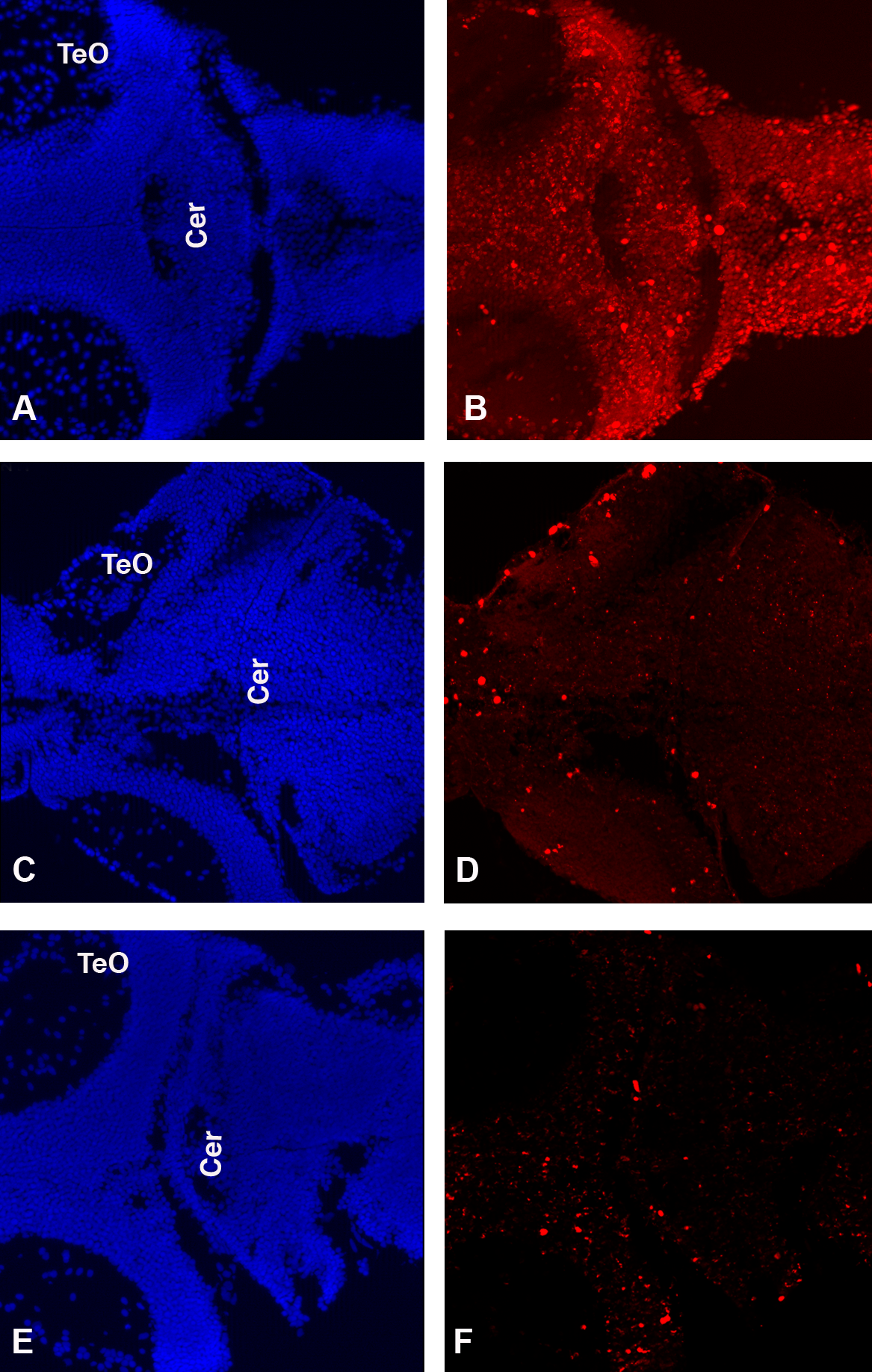

Supplement: Figure S4 — Moderate zebrafish atxn7 depletion does not induce cerebellar neuron apoptosis. Dorsal views of dissected brains from DNase-treated non-injected control (A and B) and 1 pmol mmMOzatxn7AUG (C and D) and 0.3 pmol MOzatxn7SPL morphant embryos (E and F). Anterior is to the left. DAPI staining (A, C and E) and TUNEL labelling of apoptotic cells (B, D and F). Abbreviations: TeO, tectum optic; Cer, cerebellum. (TIF) [file pone.0050705.s004.tif]

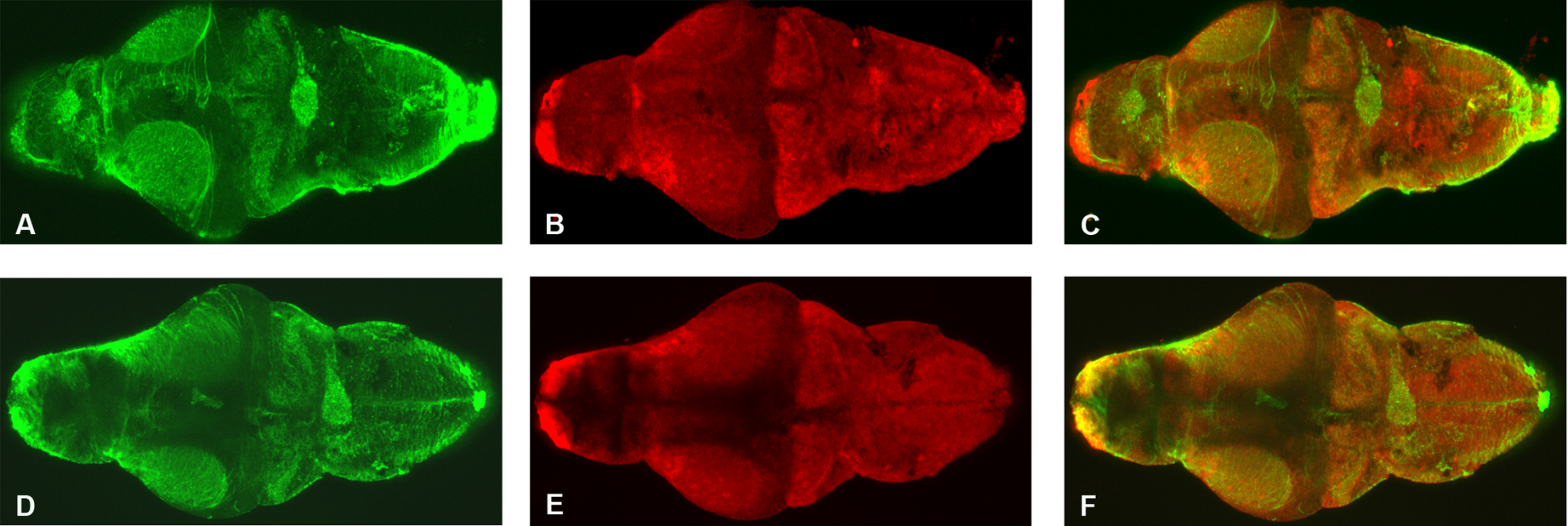

Supplement: Figure S5 — Mild zebrafish atxn7 depletion does not impair overall brain organization. Dorsal view of dissected brains from 5 dpf 1 pmol mmMOzatxn7AUG (A-C) and 0.3 pmol MOzatxn7SPL morphant embryos (D-F). Anterior is to the left. GFAP immunostaining of glial cells (A and D) and HuC immunostaining of neuronal cells (B and E). Merge images of the photographs A and B (C) and D and E (F). (TIF) [file pone.0050705.s005.tif]

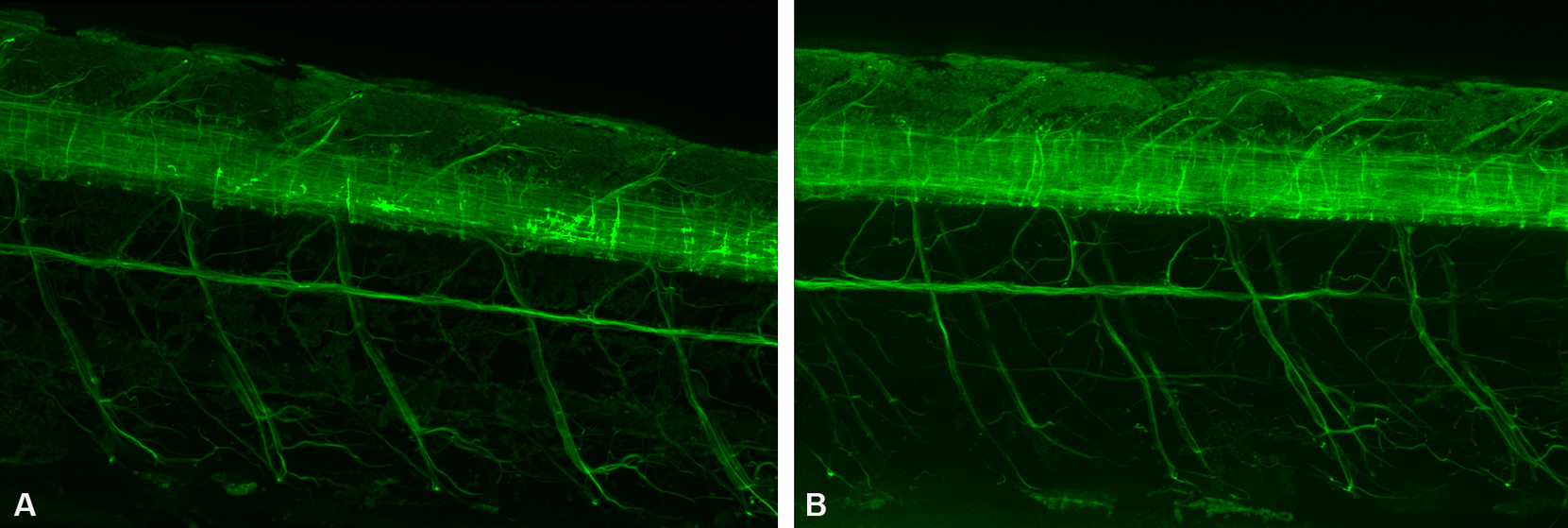

Supplement: Figure S6 — Partial zebrafish atxn7 depletion does not impair spinal cord differentiation. Lateral views of 48 hpf Tg[NBT:MAPT-GFP]zc1 transgenic embryos following injection of 1 pmol mmMOzatxn7AUG (A) and 0.3 pmol MOzatxn7SPL (B). Anterior is to the left. (TIF) [file pone.0050705.s006.tif]

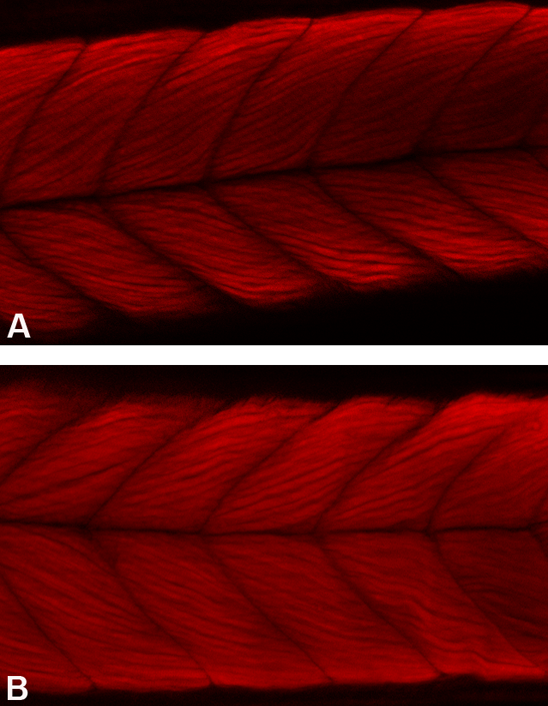

Supplement: Figure S7 — Moderate zebrafish atxn7 depletion does not impair the differentiation of trunk muscles. Lateral views of 48 hpf 1 pmol mmMOzatxn7AUG (A) and 0.3 pmol MOzatxn7SPL morphant embryos (B) following rhodamine-coupled phalloidin labelling of muscle F-actin. Anterior is to the left. (TIF) [file pone.0050705.s007.tif]
