# Supplementary material for: Requirement for Zebrafish Ataxin-7 in Differentiation of Photoreceptors and Cerebellar Neurons
Source: PLoS One. 2012 Nov 30;7(11):e50705. doi: 10.1371/journal.pone.0050705 (PMC3511343; doi:10.1371/journal.pone.0050705)
Supplement: Table S1 — Phenotypes of zebrafish atxn7 knockdown embryos. (DOCX) [file pone.0050705.s008.docx]

| Phenotypes of *zatxn7* knockdown embryos | | | | | |
| --- | --- | --- | --- | --- | --- |
|  |  |  |  |  |  |
|  |  |  |  |  |  |
|  | Percentage (No) of zebrafish embryos | | | | |
|  |  |  |  |  |  |
|  |  |  |  |  |  |
|  |  | Dead | Dead |  |  |
| Conditions | Normal | 0-6 hpf | 0-24 hpf | Deformed | Total |
|  |  |  |  |  |  |
|  |  |  |  |  |  |
| MO*zatxn7^AUG^* 1 pmol | 8.5 (14) | 24.8 (41) | 60 (99) | 31.5 (52) | 165 |
| MO*zatxn7^SPL^* 1 pmol | 9.2 (13) | 27.6 (39) | 61 (86) | 29.8 (42) | 141 |
| mmMO*zatxn7^AUG^* 1 pmol | 82.7 (129) | 14.1 (22) | 16 (25) | 1.2 (2) | 156 |
| MO*zatxn7^AUG^* 0.3 pmol | 86.7 (98) | 10.6 (12) | 12.4 (14) | 0.8 (1) | 113 |
| MO*zatxn7^SPL^* 0.3 pmol | 87.2 (109) | 8.8 (11) | 12.8 (16) | 0 (0) | 125 |
| Not injected | 90 (190) | 8 (17) | 9 (19) | 0.9 (2) | 211 |
|  |  |  |  |  |  |

**Supplementary Table 1**
